# Utilizing Behavior Change Techniques to Elicit Adherence to Clinical Practice Guidelines

**DOI:** 10.3389/fpubh.2017.00037

**Published:** 2017-03-06

**Authors:** Daniel Ferreira

**Affiliations:** ^1^Center for Sports Medicine, Concord Hospital, Concord, NH, USA

**Keywords:** continuing education, behavior change, clinical practice guidelines, best practice, orthopedic pain management

## Abstract

**Introduction:**

Two 2-day continuing education seminars were developed to address the orthopedic physical therapy deficits in Guyana. Material was presented in a way to address all stages of behavior change.

**Methods:**

Surveys evaluating preseminar and postseminar knowledge was conducted. Chart reviews to establish adherence to clinical practice guidelines were performed.

**Results:**

Preseminar surveys revealed minimal knowledge of clinical practice guidelines, which was consistent with preseminar chart review data. Postseminar data indicate improvements in both knowledge and adherence to guidelines.

**Discussion:**

A brief series of two 2-day seminars utilizing behavior change strategies to improve adherence to clinical practice guidelines shows promise for countries and regions that rely on international health volunteers to provide clinical instruction. Because this study is limited to one situation, further studies with longer follow-up in a variety of clinical settings are recommended to support generalizability of findings.

## Introduction

### Background

Chronic musculoskeletal pain (excluding arthritic pain) accounts for a third of all disability claims in the United States with over 7.6 million people on long-term disability for spine problems alone (1). Rates in developing nations are less well reported but are likely to be at similar rates. To further strain medical management resources, many developing nations are faced with a dual burden of battling infectious diseases along with non-communicable diseases such as chronic pain. Although multifactorial in nature, one factor that has been attributed to the development of chronic pain is poor management of acute and subacute pain (2–4). To assist in providing the most efficacious care, clinical practice guidelines for physical therapy (PT) interventions have been established (5–12). These evidence-based guidelines provide practitioners assistance in evaluating and treating certain common musculoskeletal pathologies. As these guidelines were designed in response to the American Physical Therapy Association’s objective of standardizing clinical care and reducing unwarranted variation, clinical practice guidelines have started emerging utilizing the most current evidence-based research (5). Despite evidence that treatment outcomes improve when utilizing clinical practice guidelines (13, 14), awareness and utilization of them is still not universal.

In Guyana, the only English-speaking country in South America, there has been historically a high reliance on Voluntary Service Overseas volunteers to administer rehabilitation services. Physical therapists in Guyana have little or no exposure to or implementation of clinical practice guidelines for the management of commonly seen musculoskeletal impairments. In fact, it has been only within the last 10 years that the government has started to invest in rehabilitation services by sending students overseas to receive PT education and later establishing a bachelor of PT program at the University of Guyana. Currently, there are eight PT in Guyana: six of whom are foreign trained (four in Cuba and two in Jamaica) and two are the first to graduate from the University of Guyana program. Of these eight PT, three hold supervisory roles and do not practice clinically on a regular basis. The remaining five PT serve the entire country and rotate through orthopedic, pediatric, and neurological settings. Therapy services are supplemented with several dozen rehabilitation technicians (RT) who undergo an 18-month program that involves introductory training in physical, occupational, and speech therapy. These RT are expected to perform evaluations and establish treatment plans as if they were PT.

In Guyana, the prevalence of non-communicable diseases is on the rise. The problems related to these diseases are often compounded by high rates of infectious disease. As the population lives longer, it is expected that chronic conditions, like chronic pain, will become more of a burden on society. Understanding and complying with clinical practice guidelines might be helpful in managing a burgeoning population of people with chronic pain—a necessary goal for Guyana to attempt to achieve.

### Behavior Change Theory

Improving compliance with utilization of clinical practice guidelines requires incorporating behavior change theory into PT education. The behavior change theory suggests that behaviors do not occur due to obstacles that impact the individual’s ability to perform desired tasks. The obstacles may be cognitive or physical in nature and vary depending on the stage along the behavior change spectrum the person is in. There are five stages of behavior change: (1) precontemplation, (2) contemplation, (3) preparation, (4) action, and (5) maintenance (15) (Table 1).

**Table 1:** **Guide to stages of change and strategies to implement**.

Stage of change	Problem faced at this stage	Strategies
Precontemplative	Unaware of desired behaviors	Consequence-based arguments focusing on desirability and likelihood (use social stats)
Contemplative	Perceptions that others do not think they should change (prescriptive norms); or perceptions others are not performing given behavior (descriptive norms)	Encourage more weight on own beliefs. Help change social norms (get others who support behavior involved)
Preparation	Perception of poor ability	Provide opportunities for success with rehearsal and modeling
Action	Motivation and opportunity	Utilize point-of-action cues (like prompts in medical record) and explicit planning
Maintenance	Relapse to prior behaviors once prompts are removed	Provide encouragement and positive feedback to practitioner. Reward good desired behavior

The first stage, precontemplative, is where the individual is unaware of the desired behavior and therefore does not perform it. People in this stage benefit from knowledge dissemination and actions that draw awareness to the behavior and its consequences. Consequence-based arguments work well in this stage of change (16).

The next stage is contemplation where the individual is aware that there is a problem, but is unclear about strategies and may be limited by perceived social norms. Both descriptive (a person’s perception of whether others perform the given behavior) and prescriptive (person’s perception of what other people think that they should do) norms can influence peoples’ behavior in this stage. Overcoming these norms is crucial to move beyond contemplative to preparation stages of change (17).

The crucial element of the third stage, preparation, is the need to provide self-efficacy training to ensure that individuals feel comfortable with their abilities to perform the desired behavior. Even with knowledge of the consequences of the behavior—and addressing both prescriptive and descriptive norms—if there is low perceived ability, there is less likelihood that the behavior planning will turn into the fourth stage, action. Therefore, removing obstacles to performing action is necessary in this stage. Self-perceived limitations of skill can be overcome with modeling and rehearsal (18). Transfer from preparation to action has been shown to be optimal when both positive and negative scenarios are presented, when practice included trainee-generated scenarios and instructed to set goals, and when participants’ supervisors were also trained (19).

Action is the stage when the desired behavior is attempted. Ways to facilitate this stage are to provide prompts and explicit planning strategies. In the clinic, this means providing opportunities to be reminded of the need to adhere to the clinical guidelines (possibly in the chart, etc.) and creating scenarios where the treatment planning is concretely determined to optimize treatment. Prompts provided at points of decision have been shown to be effective in modifying a variety of behaviors (20–22).

Once action occurs, the person moves into the last stage, which is maintenance. Maintenance is the continuation of the action after the prompting has stopped. In this stage, it is important to provide support to the individual along with positive feedback to ensure continued compliance.

Similar to health care practitioners and their patients, far too often educators treat all learners as if they are in the precontemplative stage by providing information (almost always in a consequence-based argument format) regardless of assessing the stage of change they may be in. Recognizing that individuals may be in different stages of change is necessary to effectively elicit the desired behavior outcomes. Instructors simply providing information on the best practice guidelines without addressing barriers (real or perceived) has been shown to be ineffectual and not an ideal mechanism to elicit change in treatment practice.

### The Process

A Peace Corps Response volunteer with a Doctor of Physical Therapy degree, and 10 years of clinical experience was placed at the Ministry of Health’s rehabilitation department to help supervise PT students and work with the RT and establish whether there were any areas of deficit in their orthopedic skills. Although he was there, he carried out a needs-assessment that indicated a lack of awareness about clinical practice guidelines and a heavy reliance on passive modalities (especially ultrasound). In addition, evaluations did not typically include special tests that might help guide therapists in their clinical decision-making. From that assessment, a proposal for continuing education was developed, which would utilize a volunteer instructor.

In this case report, therapist knowledge of clinical practice guidelines will be discussed, and the outcomes of two 2-day symposia, utilizing behavior change techniques to influence evidence-based best practice, will be described.

## Methods

### Symposia Development

Knowledge and use of clinical practice guidelines for common musculoskeletal orthopedic conditions (lower back, neck, hip, knee, and ankle/foot pain) was determined to be the most critical continuing education need. Therefore, two 2-day symposia were created: Symposium I and Symposium II. The symposia were planned so that Symposium II would take place 2 months after Symposium I.

Evidence suggests that dissemination-only training interventions have little to no effect (23) since these interventions are only useful for people in the precontemplative stage of change. Strategies that also address other stages of change may be more effective. Therefore, the symposiums involved not only presentation of materials but also an opportunity to practice skills and problem-solve scenarios. The objectives of the symposia were threefold.

First, it was designed to draw attention to clinical practice guidelines. This mirrors the precontemplative stage of the behavior change model. Practitioners were provided with consequence-based arguments on the importance of providing optimal care. Second, social norms were addressed discussing expectations of the therapists and patients. Addressing social norms is important to draw attention to best practice compliance among their peers to allow those in the contemplative stage to consider the need for changing their own behavior/practice. And the last objective was to provide the opportunity to actually rehearse and model the behaviors and techniques necessary to adhere to the clinical practice guidelines. Thus, the symposium was broken into three parts, a lecture element where the material was presented, breakout groups where scenarios and current practice were discussed and concerns were addressed, and a hands-on lab section where the volunteer demonstrated the techniques and then had clinicians practice. Continued practice was encouraged once the symposium was finished.

## Symposium I

The first symposium focused on introducing clinical guidelines for the five orthopedic conditions. This symposium was composed of approximately 50% lecture and 25% for breakout groups and hands out sections. In this manner, drawing learners’ attention to the clinical practice guidelines and providing them information *via* consequence-based arguments on their importance was utilized for learners’ in the precontemplative stage. As was seen with the presymposium survey, most practitioners were in this precontemplative stage. If the survey was aware of the guidelines but did not adhere to them, then the initial emphasis may shift to another stage with less time spent on the basic information. However, as mentioned, these practitioners did require this information and thus were provided with consequence-based arguments on the importance of providing optimal care.

Instruction did not end at this level, however, as social norms were addressed by discussing expectations of the therapists and patients. This was done to draw attention to best practice compliance among their peers to allow those in the contemplative stage to consider the need for changing their own behavior/practice. As their most immediate peers were also not compliant, a larger cohort (i.e., international) was utilized to illustrate that the *majority* of their peers did, in fact, adhere with the evidence-based clinical recommendations. Therefore, a portion of the lecture was utilized to discuss to social norms. This discussion carried over to breakout groups to discuss among each other case studies and application of the newly learned materials.

The last portion of the first symposium was to provide the opportunity to actually rehearse and model the behaviors and techniques necessary to adhere to the clinical practice guidelines. This hands-on portion contained instruction and performance of the actual techniques recommended by the clinical guidelines. Emphasis of this section was on the manual interventions as these require the most intensive support and guidance for proper technique, review of red flags, and comfort to perform independently. Approximately 4 h were spent with clinicians practicing on each other the variety of manual techniques. In addition to proper technique, increasing comfort also helps to reduce perceived barriers to implementation of recommendations and thus can help those practitioners in the action stage remain adherent or allow those in the preparation phase move to action.

Clinicians were encouraged to continue to practice on each other as well as start utilizing techniques and guidelines in their daily practice. In the 2 months between symposia, the instructor was available *via* email. He was also available in-person for instruction and modeling for therapists located in the capital of Guyana. Clinicians were encouraged to contact the instructor if they had further questions or needed clarification during the interim.

## Symposium II

The second symposium reviewed the clinical practice guidelines briefly (~15% of time) but spent far more time honing clinical skills (~60% of time) and allowing for breakout sessions to review cases (~25% of time).

### Pretests and Posttests

Two pen-and-paper tests of knowledge were developed by author and were reviewed by another US-based therapist with 10 years of clinical and academic experience. Input was provided, and modifications were made to improve clarity. Each test included five clinical scenarios: one for each of the five clinical guidelines [neck (6), low back (8), hip (7, 9), knee (11), and ankle/foot (5, 12)]. Each scenario was followed by five questions that were created to assess knowledge. The clinical scenarios were designed to elicit information about clinical decision-making. Multiple-choice options for each question were designed to offer a wide variety of treatment options, but only one held the proper choice advocated by the clinical practice guidelines. For each scenario, there was at least one treatment option that included the inappropriate use of ultrasound.

After gaining informed consent, participants were randomly assigned to complete one of the pen-and-paper tests prior to Symposium I. After Symposium II, they completed the alternate test.

Participants were instructed to make decisions about the case scenarios to determine appropriate treatment options. Survey results would be evaluated for correct responses and to evaluate deviation of scores between the two variations of the test.

The second part of the survey evaluated demographics of the therapists such as years of experience, job type (RT vs PT), and national or foreign education.

### Chart Reviews

Chart reviews were performed before and after symposia to evaluate clinician treatment selection. Two charts from each clinician currently working in an orthopedic setting (*n* = 16) were reviewed for each of the five clinical presentations (10 total charts reviewed). Charts reviewed were from patients who initiated treatment after the first symposium. Ten charts were also reviewed from patients seen prior to the first symposium and were to serve as a comparison.

Chart reviews explored whether documentation of clinically important questions were asked during history taking, whether appropriate special tests that assist therapists in correctly classifying patients into certain treatment groups were performed, and whether treatment selection matched clinical assessment. Each element was given a score of 0 (absent) or 1 (present). Charts were graded from 0 to 3. As a separate factor, the prevalence of ultrasound treatment was also evaluated.

## Results

A total of 21 clinicians attended both symposia, and data were collected from those participants (Table 2).

**Table 2:** **Clinician demographics**.

Total respondents (*n*)	21 (18 females/3 males)
Percentage with physical therapy degree	38% (*n* = 8)
Average years of practice	12.8
Percentage physical therapists trained overseas	75% (*n* = 6)
Percentage of clinicians with at least 1 year rotation in orthopedics	95% (*n* = 20)
Percentage of clinicians currently work in orthopedic setting	76% (*n* = 16)

### Awareness of Guidelines

#### Data from Pretest and Posttest

Presymposia results indicated that clinicians were unaware of clinical guidelines as correct selection of proper treatment options was infrequent. In total, 27–30% of participants were able to correctly recommend treatment options for neck pain, 18–20% for back pain, 30–36% for hip pathology, 45–50% for knee pain, and 36–40% for foot and ankle pain (Table 3). Individually, only one respondent was able to answer all five questions correctly. The other 20 respondents averaged 30% correct responses.

**Table 3:** **Accuracy of responses to clinical decision-making questions**.

	% Correct presymposium	% Correct postsymposium	% Change in correct response
**Survey 1**	***n* = 10**	***n* = 11**	

Question 1 (neck)	30% (*n* = 3)	82% (*n* = 9)	200%
Question 2 (back)	20% (*n* = 2)	71% (*n* = 8)	300%
Question 3 (hip)	30% (*n* = 3)	91% (*n* = 10)	233%
Question 4 (knee)	50% (*n* = 5)	91% (*n* = 10)	100%
Question 5 (ankle/foot)	40% (*n* = 4)	82% (*n* = 9)	125%

**Survey 2**	***n* = 11**	***n* = 10**	

Question 1 (neck)	27% (*n* = 3)	70% (*n* = 7)	133%
Question 2 (back)	18% (*n* = 2)	60% (*n* = 6)	150%
Question 3 (hip)	36% (*n* = 4)	80% (*n* = 8)	100%
Question 4 (knee)	45% (*n* = 5)	90% (*n* = 9)	80%
Question 5 (ankle/foot)	36% (*n* = 4)	80% (*n* = 8)	100%
Total survey correct responses	33% (35/105)	80% (84/105)	140%

Postsymposium surveys revealed an improved understanding of clinical guidelines (see Table 3). The percentage increase in correct responses ranged from 80 to 300%. The lower percentage increase appears to be related to a ceiling effect based on a higher initial correct response rate.

#### Chart Review

This improvement in knowledge did appear to correspond with a carryover to treatment as review of treatment choice revealed a significant improvement in utilization of evidence-based treatment options while use of ultrasound was concurrently reduced (Table 4). Of note, treatment compliance did not increase consistently across the five body parts investigated. Knee treatment compliance rated at 75%, while neck intervention compliance was at 32%. This may be seen as a result of a more uniform treatment regime for treating knee pain as opposed to a diverse treatment protocol for the variety of neck problems.

**Table 4:** **Chart review findings**.

	Presymposium	Postsymposium	Percent change
Correct use of special tests			
Neck	6% (2/32)	38% (12/32)	500%
Back	3% (1/32)	47% (15/32)	1,400%
Hip	19% (6/32)	63% (20/32)	233%
Knee	13% (4/32)	32% (10/32)	150%
Foot/ankle	6% (2/32)	32% (10/32)	400%
Clinically important questions asked during history			
Neck	19% (6/32)	38% (12/32)	100%
Back	25% (8/32)	50% (16/32)	100%
Hip	13% (4/32)	41% (13/32)	225%
Knee	19% (6/32)	47% (15/32)	150%
Foot/ankle	22% (7/32)	53% (17/32)	143%
Treatment matching clinical assessment			
Neck	0% (0/32)	32% (10/32)	–
Back	0% (0/32)	50% (16/32)	–
Hip	0% (0/32)	63% (20/32)	–
Knee	6% (2/32)	75% (24/32)	1,100%
Foot/ankle	7% (4/32)	38% (12/32)	200%
Use of ultrasound	94% (30/32)	38% (12/32)	60%

Qualitatively, documentation of charts overall saw improvement (coherence, flow, and reasoning). Clinically important history questions increased markedly but still were occurring in less than 50% of chart reviews. Similarly although special tests were being performed more frequently after the symposium, they were not consistent as hip and back special tests that were performed at quite a higher rate than neck, knee, and foot/ankle. While clinical assessments were still less than optimal, they also saw improvement. Use of appropriate orthopedic special tests also improved.

## Discussion

This report shows that even a small and modest study can provide information that can contribute to improving health professions training that ultimately improves clinical care. However, the study does have some limitations that must be acknowledged. Limitations include a small sample size, a short follow-up, as well as not evaluating whether the clinicians did any further research on their own that may have skewed postsurvey scores. The small sample size could not be overcome due to the dearth of clinicians in Guyana. The short follow-up does draw to question whether there will be long-term carryover in knowledge although it was promising that both knowledge and behavior were changed. A model that includes continued prompts, communication with volunteer, and “refresher” courses is likely to improve chances of maintenance of desired behavior.

Also it is unclear what elements of the symposia (lecture, discussion of patient scenarios, or rehearsal and hands-on practice) were the most effective. There is also the possibility of the Hawthorne Effect impacting performance; where clinicians knew that their performance would be monitored and therefore chose improved treatment options. However, if this is the case, it bolsters the argument for need for continued cuing and feedback as part of the maintenance phase of change. Another limitation is with survey design. As there were two separate surveys with similar but not identical case scenarios, it is possible that one was easier than the other, which might have skewed the results. However, regardless of this overestimation of the percent improvement in correct responses, there was still a significant increase indicating at least a degree of retention of knowledge, which corresponded with increase in proper treatment choice noted in chart review. Finally, Guyana being an English-speaking country made provision of supplemental materials and electronic communication easier than it would be for a volunteer in a foreign language-speaking nation.

From this study, it was evident that knowledge about clinical practice guidelines was sparse among both PT and RT in Guyana. Promisingly, it appears that brief bouts of concentrated in-services focusing on addressing the stages of behavior change supplemented with electronic communication available appear to improve knowledge of best practice. It also appeared to improve adherence to PT clinical guidelines at the 2-month follow-up. In addition, treatment strategy was shown to be modified with a single bout of continuing education and addressing several stages of behavior change. This suggests that that volunteer-based education platforms focusing on areas of greatest educational needs may positively impact clinical decision-making and treatment plans.

This model may be most appropriate for countries and regions with the least amount of basic resources and knowledge as there was a large area of improvement that was available, making significant change easier to elicit than if in a situation where there had been some knowledge but inappropriate treatment adherence and ingrained poor practice interventions. Having no knowledge may have actually been a positive as it allowed for tabula rasa approach where adherence without conflicting strategies may have been easier. It is unclear whether it was a simple exposure to non-conflicting information or whether intervention to modify behavior elicited the change seen in treatment intervention.

Promoting behavior change through proven strategies based on an individual’s readiness to change is often an underutilized strategy when providing in-services and educational seminars but may be necessary to optimize best practice utilization. Understanding that not all health care practitioners are limited by a lack of knowledge but rather by possible knowledge, social, or physical constraints that need to be addressed is an important first step in shifting the focus of educational interventions. Providing educational materials that meet the learners’ needs is a crucial element that this article discusses. Once educational content matches learners’ needs, the transfer from abstract information to practical application is possible.

Utilizing international partnerships has long been a cornerstone in provision of health care education and support. As many developing nations do not have the resources to provide this support, these partnerships remain important for delivery of health care. Based on educational research, providing simple information-only learning material is not very efficacious. Therefore, there may need to be a paradigm shift in how educational support is provided. This study suggests that utilizing the behavior change model to evaluate where learners are in stages of change may provide benefit in promoting evidence-based practice awareness and adherence. It also strengthens the argument for short-term international partnerships where skilled clinicians can provide brief concentrated bouts of education concentrating on the areas of greatest need.

Despite limitations, this case report suggests that there may be positive effects of short-term continuing education interventions utilizing behavior change techniques supplemented with electronic communication to improved clinical practice guidelines adherence and eliciting practice change. International collaborations that can provide educational materials targeted at the level of behavior change of the learners may increase best practice compliance. Further studies that can control for confounding variables with a larger and more diverse sample size are needed before recommending the implementation of behavior change strategies universally. Considering readiness to change while providing education does hold promise to better address clinical guideline adherence.

## Author Contributions

The author confirms being the sole contributor of this work and approved it for publication.

## Conflict of Interest Statement

The research was conducted in the absence of any commercial or financial relationships that could be construed as a potential conflict of interest.
